# A case report of Cogan's syndrome with recurrent coronary stenosis

**DOI:** 10.3389/fcvm.2024.1451113

**Published:** 2024-09-12

**Authors:** Gao Na, Zhang Nan, Meng Jingjing, Pan Lili

**Affiliations:** ^1^ Department of Rheumatology, Capital Medical University Affiliated Anzhen Hospital, Beijing, China; ^2^ Department of Radiology, Capital Medical University Affiliated Anzhen Hospital, Beijing, China; ^3^ Department of Nuclear Medicine, Capital Medical University Affiliated Anzhen Hospital, Beijing, China

**Keywords:** Cogan’s syndrome, variable vasculitis, recurrent coronary stenosis, case report, coronary angiography

## Abstract

Cogan's syndrome (CS) is recognized as a form of variable vasculitis. This report presents the case of a middle-aged woman experiencing recurrent coronary artery stenosis, accompanied by a history of non-syphilis keratitis, vestibular auditory symptoms, and venous thrombosis. Positron emission tomography/computed tomography revealed an elevated uptake of (18)F-fluorodeoxyglucose in the subclavian artery, common carotid artery, aortic arch, and thoracic aorta. A diagnosis of Cogan's syndrome was made. The aim of this study was to increase clinicians’ awareness of the vascular manifestations in CS and to emphasize the importance of thorough history taking. CS should be included in the differential diagnosis when patients present with recurrent coronary artery stenosis.

## Introduction

Cogan's syndrome (CS) is a rare autoimmune disease, often categorized as a variable vasculitis, and is most commonly diagnosed during the third or fourth decade of life, with a similar prevalence in women and men. The presence of non-syphilitic keratitis and vestibuloauditory symptoms within 2 years is indicative of typical CS (1). Aortitis may affect approximately 10% of CS patients (2). This case report highlights a rare case of coronary artery stenosis in the context of CS, underscoring the need for vigilance in clinical practice.

## Case description

**Figure 1:**
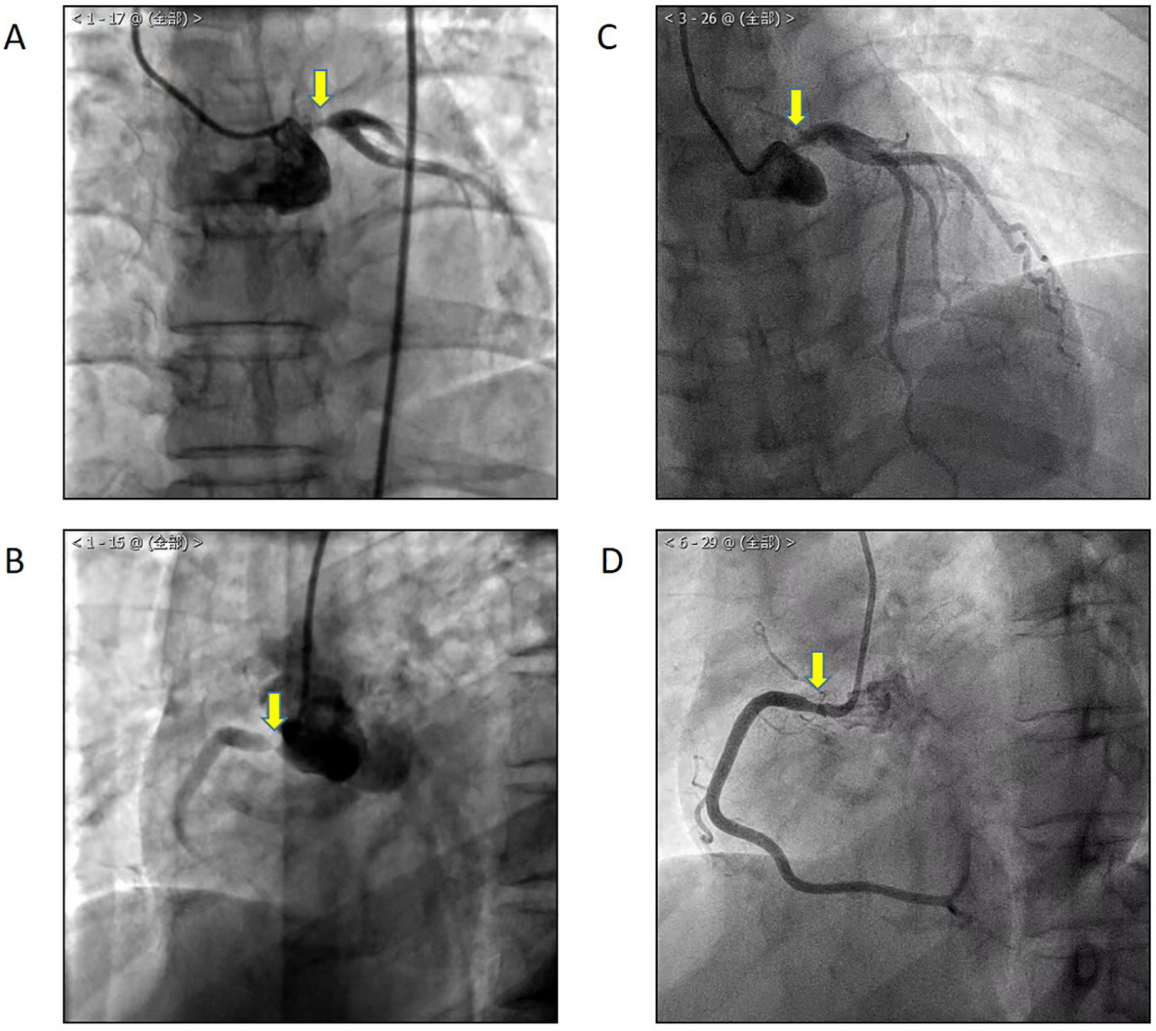
In 2020, CAG showed LM **(A)** and RCA **(B)** stenosis. One year after percutaneous coronary intervention, the patient suffered from chest tightness and a shortness of breath again, and CAG showed LM **(C)** and RCA **(D)** in-stent restenosis.

A 49-year-old female patient was admitted in 2021 with a complaint of chest tightness and a shortness of breath, symptoms that had been present for a year and were exacerbated by satiety and physical activity. She was diagnosed with non-ST segment elevation myocardial infarction with stenosis of the left main trunk (LM) and right coronary artery (RCA) (Figure 1) and had undergone percutaneous trans-luminal coronary angioplasty and intervention, followed by secondary prevention and treatment for coronary heart disease. Recently, the aforementioned symptoms have re-emerged. The patient reported no fever, rash, oral or genital ulcers, erythema nodosum, or intermittent claudication.

**Figure 2:**
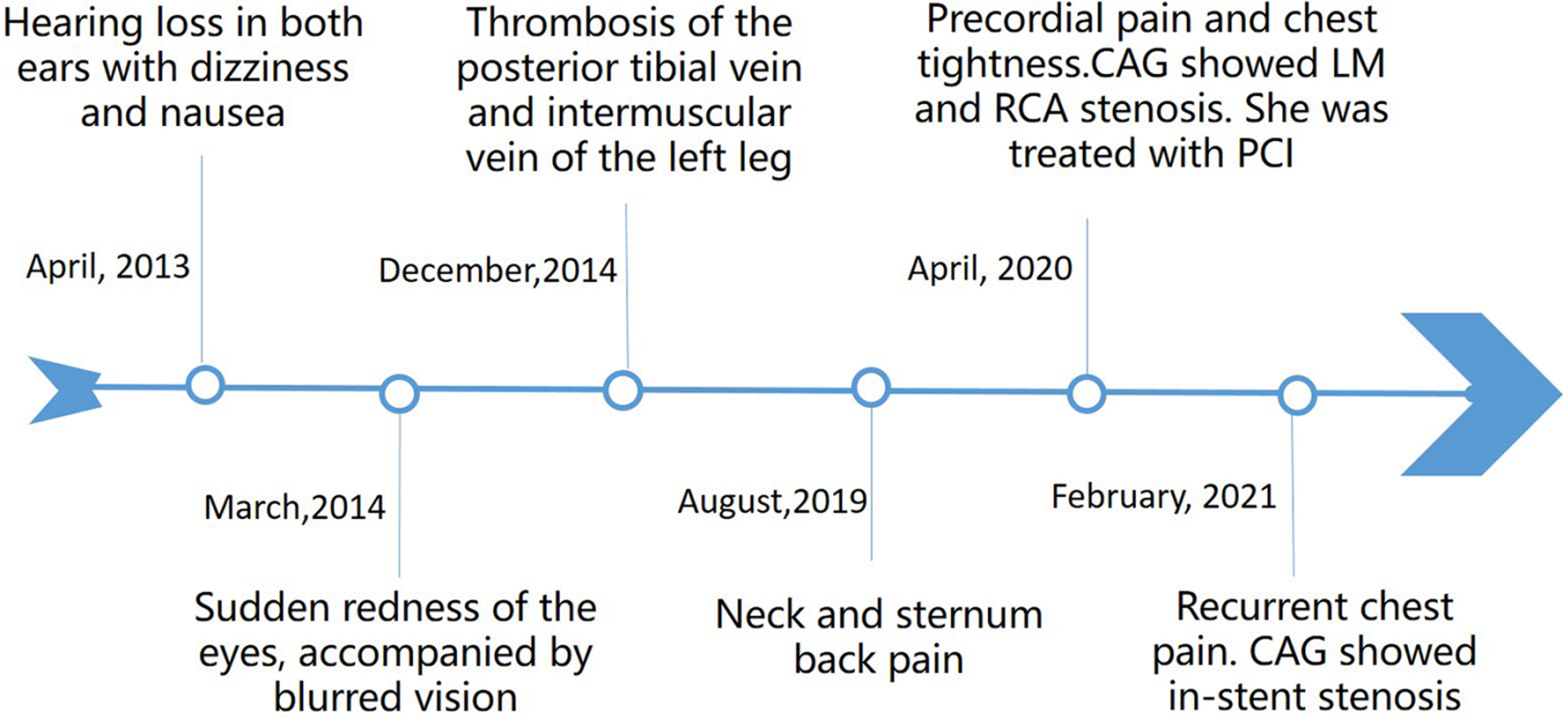
A timeline with the relevant data from the episode of care.

A comprehensive medical history (Figure 2) revealed a history of sudden hearing loss, vertigo, and nausea, with recurrent episodes from 9 years ago. She had experienced episodes of redness and blurred vision in both eyes 8 years ago, which improved with antibiotic and corticosteroid treatment. Over the years, she had also suffered from symptoms of vertigo and blurred vision, accompanied by photophobia, which were alleviated with glucocorticoid use, although her hearing progressively declined. In addition, she had a history of limb venous thrombosis, which responded to anticoagulant treatment. There were no special family and psychosocial histories and genetic disorders.

Physical examination findings included a nebula on the lateral edge of the cornea in both eyes, decreased hearing in both ears, and reduced pulsation in the radial, brachial, and dorsal pedal arteries, particularly on the right side. Vascular murmurs were detected in the auscultation areas of the bilateral carotid, subclavian, renal, and iliac arteries and abdominal aorta.

In laboratory tests, the erythrocyte sedimentation rate was 97 mm/1 h, C-reactive protein was above 20 mg/L, complement C3 was 1.72 g/L, complement C4 was normal, immunoglobulin and serum immunoglobulin G4 were normal, and IL-6 was 49.67 pg/ml. Antinuclear antibody profile, neutrophil cytoplasmic antibody, antiphospholipid antibody, interferon-gamma release assay for mycobacterium tuberculosis, syphilis antibody, and tumor marker tests were all negative, and n-terminal prohormone B-type natriuretic peptide was 185.00 pg/ml.

**Figure 3:**
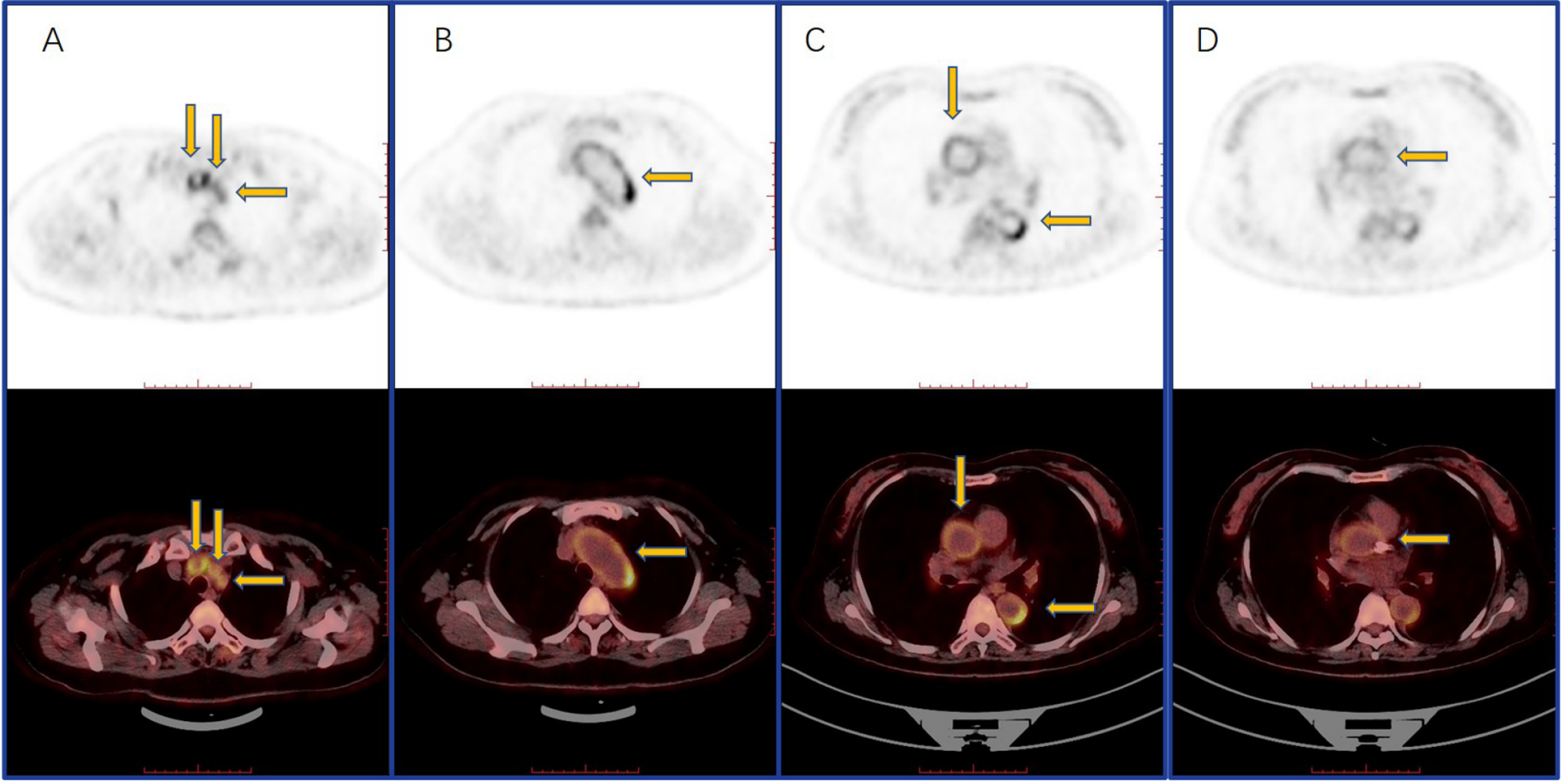
PET-CT showed an increased circular and linear 18F-FDG uptake in the subclavian and common carotid arteries **(A)**, aortic arch **(B)**, thoracic aorta **(C)**, and coronary **(D)**.

Upon review, coronary angiography (CAG) showed in-stent restenosis (Figure 1). Echocardiography revealed thinning of the left ventricular inferior wall basal segment, mild aortic regurgitation, and decreased left ventricular diastolic function. Positron emission tomography/computed tomography (PET/CT) demonstrated an increased uptake of (18)F-fluorodeoxyglucose (18F-FDG) in the aorta and its primary branches, as well as in the coronary arteries (Figure 3).

Given the patient's ocular, auditory, vestibular, and vascular symptoms, a diagnosis of CS was confirmed. We conducted a differential diagnosis to rule out the following conditions: (1) Takayasu’s arteritis: the patient presented with multiple arterial stenoses, necessitating the consideration of Takayasu’s arteritis. However, the occurrence of stromal keratitis and sensorineural hearing loss did not align with the typical manifestations of this condition. (2) Antiphospholipid syndrome: the patient's history of recurrent thrombosis and multiple arterial stenoses raised the possibility of antiphospholipid syndrome. However, the absence of phospholipid antibodies in all tests negated this diagnosis. (3) Behçet's syndrome: despite the need to consider Behçet's syndrome given the patient's symptoms, the lack of oral and genital ulcers, erythema nodosum, and other characteristic skin changes, as well as the absence of ocular involvement, did not fulfill the criteria for this diagnosis.

The patient was managed conservatively without undergoing repeat percutaneous coronary intervention (rePCI) or bypass surgery involving the internal mammary artery (IMA) to the left anterior descending artery (LAD). The patient was managed conservatively with aspirin, clopidogrel, and isosorbide mononitrate. In tandem with this approach, we initiated a therapeutic regimen that included a combination of corticosteroids and cyclophosphamide to address vasculitis at the same time. The treatment began with prednisone at a dosage of 50 mg once daily, complemented by intravenous cyclophosphamide at 0.4 g administered biweekly. Subsequently, we implemented a gradual reduction in prednisone and transitioned to methotrexate at 15 mg weekly to alleviate the potential long-term adverse effects associated with cyclophosphamide.

The patient maintained remission over the subsequent 3 years, with no recurrence of chest pain, stable vision and hearing, and no further episodes of venous thrombosis. Laboratory tests and coronary angiography remained stable. The patient had good adherence and tolerability to the intervention, and no adverse or unanticipated events occurred.

## Discussion

This case highlights the importance of considering Cogan's syndrome in the differential diagnosis for patients presenting with recurrent coronary artery stenosis. The patient's history of ocular abnormalities, hearing loss with vertigo, and subsequent development of multiple artery stenoses and venous thrombosis, along with PET/CT (a valuable tool in the diagnostic and therapeutic management of vasculitis due to its comprehensive and detailed imaging capabilities) findings of a high 18F-FDG uptake in the aorta and its branches, supports this diagnosis. We conducted a differential diagnosis to rule out other conditions. The limitation was that the patient was not assessed using intravascular ultrasound (IVUS) or fractional flow reserve (FFR) during the coronary angiography. A previous study (3) has suggested that the histological presence of inflammatory cells in the vessel walls of CS patients may be indicative of the disease's vasculitic nature, which can be managed with immunosuppressive therapy (4). When faced with such cases, clinicians are encouraged to conduct thorough history taking and consider CS as part of the differential diagnosis.

## Data Availability

The original contributions presented in the study are included in the article/Supplementary Material; further inquiries can be directed to the corresponding author.
